# Field-programmable encoding for address-event representation

**DOI:** 10.3389/fnins.2022.1018166

**Published:** 2022-12-05

**Authors:** Prafull Purohit, Rajit Manohar

**Affiliations:** Electrical Engineering, Yale University, New Haven, CT, United States

**Keywords:** address-event representation (AER), FP-AER, asynchronous, event-driven, arbitration, neuromorphic, event-based computing, event-based communication

## Abstract

In conventional frame-based image sensors, every pixel records brightness information and sends this information to a receiver serially in a scanning fashion. This full-frame readout approach suffers from high bandwidth requirements and increased power consumption with the increasing size of the pixel array. Event-based image sensors are gaining popularity for reducing the bandwidth and power requirements by sending only meaningful data in an event-driven approach with the help of address-event representation (AER) communication protocol. However, the event-based readout suffers from increased latency and timing error when the number of pixels with an event increase. In this paper, we introduce a new field-programmable AER (FP-AER) encoding scheme which offers benefits of both frame-based and event-based approaches. The readout design can be configured “in the field” using configuration bits. We also compare the performance of the proposed design against existing AER-based approaches for imaging applications and show that FP-AER performs best in both scanning and event-based readout.

## 1. Introduction

Conventional frame-based imagers use a data-centric approach in which all pixels in a sensor measure the incident light for a pre-defined time as charge, voltage, or current. This value is then digitized at some place in the readout chain and values from all the pixels are sent to the acquisition system (receiver). With an increase in the number of pixels over time, sending this data to the receiver requires more time and energy due to an increase in the total pixel count, as well as the energy and delay cost per pixel. As a result, improvements are generally made on increasing readout speed with increased power consumption or decreasing power consumption with a slower data rate.

**Figure 1:**
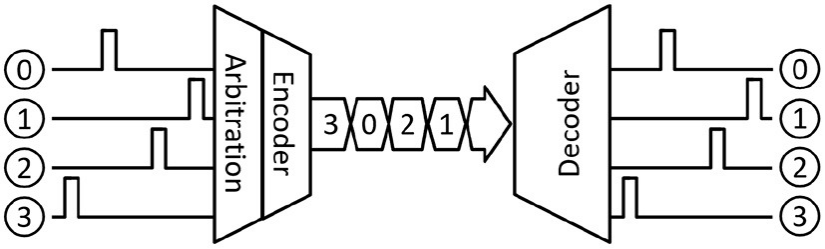
AER communication.

In contrast, event-driven imagers use an asynchronous approach in which individual pixel measure local changes in the incident light intensity (brightness) and produces an asynchronous event. The events are then communicated to the acquisition system (receiver) through a shared output bus in the form of address-event representation (AER) (Liu et al., 2014). In typical AER-based communication systems, as shown in Figure 1, an address encoder waits for an incoming event and multiplexes the event location as an encoded address on the output bus. When multiple events arrive in a short time, an arbitration mechanism selects one of them and sends its address on the output bus. The event timing is represented by the relative position of the event address in the output stream. With an increase in the number of events, a typical AER-based readout becomes less power efficient, and readout latency increases (iniVation AG, 2020).

With advancements in microelectronic technologies, two different scaling scenarios for imaging devices can be observed: pixel-level scaling and chip-level scaling. In the case of chip/package level scaling, pixel size remains the same but imager size increases by adding more pixels to the array. In such cases, an AER-based readout is beneficial because it can still process the event sparsely whereas a frame-based readout would need to read additional pixels for the same information content. In the case of pixel-level scaling, a reduction in pixel size results in increased pixel density in a given area. A physical “hit" will likely create an event “cloud" and increase the number of events in the region. In such cases, an AER-based readout becomes less power efficient, and the readout latency increases (iniVation AG, 2020) while a frame-based readout can quickly process the group of events without requiring multiple communication handshakes on the output bus. In summary, frame-based imagers focus on full-frame readout with limited capabilities for data compression and region-of-interest readout. On other hand, an event-based imager normally focuses on sparse readout by sending only pixels with an event. Because of this focused approach, the readout architecture is highly customized to support either frame-based or event-based readout. In this paper, we present a new field-programmable address-event encoding scheme that is suitable for both frame-based (scanning) and event-based (arbitrated) imaging applications. The arbitration mechanism is implemented based on the binary tree with each arbitration module modified to support token-ring configuration. The design configures into event-mode (arbiter tree) or frame-mode (token-ring) based on the user-programmed control bits. In event mode, the design operates like a greedy tree arbiter (Boahen, 2004). In frame mode, the design operates as a linear token ring (Imam and Manohar, 2011) and a single token travels through a token ring to provide fast, multiplexed access to the shared output bus. As shown in **Figure 3C**, the design also supports a hybrid mode where lower stages are configured as a greedy tree and the topology ends in a token-ring at the highest level, offering configurable granularity of parallelism at lower levels. This allows a user to configure readout topology and efficiently support different cluster sizes. In such configurations, localized events can be treated as a cluster and handled quickly while offering each cluster access to the output in a fair manner through a higher-level ring.

The rest of the article is organized as follows. Section 2 provides a brief introduction to previously designed encoding schemes for AER and discusses their implementation. Section 3 describes a few imaging modes, scientific applications for each of the modes, and our motivation to combine commonly used encoding schemes. Details of the proposed encoding scheme and circuit implementation are provided in Section 4. Section 5 presents simulation results and comparisons with existing encoding schemes, and we conclude in Section 6.

## 2. Review of AER topologies

Address-event representation (AER) is an event-driven, neuromorphic inter-chip encoding and communication protocol originally proposed to communicate the location and timing information of sparse neural events between neuromorphic chips (Sivilotti, 1991). Since Sivilotti (Sivilotti, 1991) and Mahowald (Mahowald, 1992) first proposed the use of AER for communicating spikes, it has been used in many silicon retina and bio-inspired vision sensors (Culurciello et al., 2001, 2003; Boahen, 2004; Lichtsteiner et al., 2008; Posch et al., 2010; Brandli et al., 2014). A similar event-driven approach was developed for imaging devices such as pixel array detectors (PADs) and monolithic active pixel sensors (MAPS) for scientific applications (Williams et al., 2014; Margarit, 2016). As shown in Figure 1, a typical AER-based sender implements two functions: encoding and arbitration. The encoder waits for an event and encodes the event identity based on its location. This encoded “address-event” is then sent across the output bus as they occur, preserving the timing information. When two or more events arrive simultaneously, an arbitration mechanism is used to avoid collision and determine which event is communicated first on the output bus. A queuing mechanism is introduced to allow other events to wait for their turn. This queuing can be at the source (per pixel), shared (per AER encoder), or a combination of the two (Liu et al., 2014). A receiver, on the other hand, receives the encoded “address-event” and decodes the event location based on the received event identity. This decoded event identity is then used to generate an event on the output in the same order as the received “address-events.” A variety of schemes have been proposed in the literature for designing an AER-based communication interface. Purohit and Manohar (2021) provides a summary of recent AER schemes. These schemes differ in the mechanism they use for resolving conflicts when two or more events occur simultaneously on the input channels, and how the event identity is encoded (Liu et al., 2014). Based on the arbitration mechanism and address encoding, two common approaches to designing an AER-based interface have emerged.

### 2.1. Tree-based arbitration

**Figure 2:**
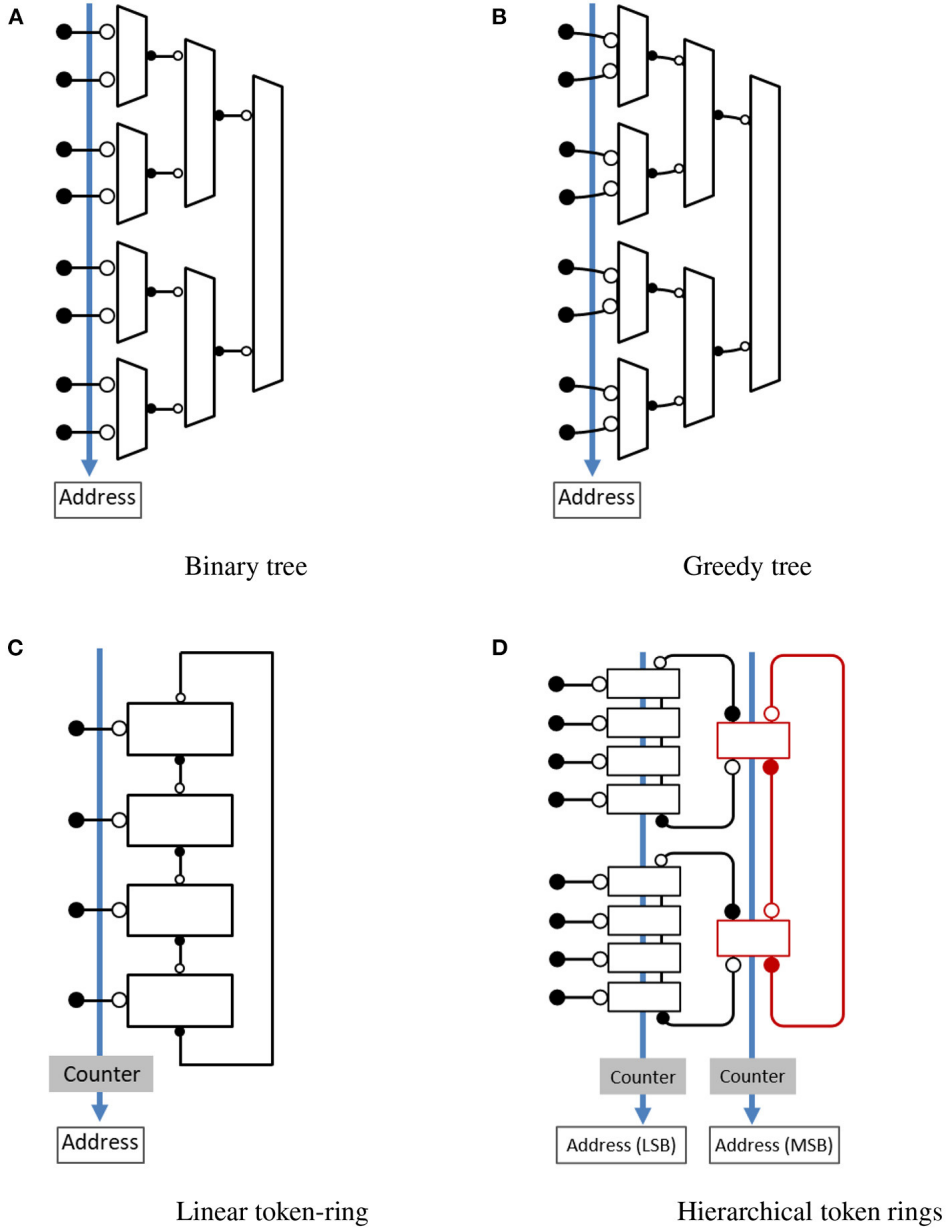
Address-event encoding schemes. ⊸ denotes the passive end of the channel and −• denotes the active end of the channel. **(A)** Binary tree. **(B)** Greedy tree. **(C)** Linear token-ring. **(D)** Hierarchical token rings.

Tree-based arbitration was first proposed by Mahowald (1992) and Lazzaro et al. (1993) for use in silicon retina but this approach has been used in many event-based image sensors. The simplest and most popular implementation of this approach, as shown in Figure 2A, is based on a hierarchical structure of 2-input arbiters arranged in a tree configuration. When a request arrives on one of the inputs, it travels to the top where a root arbiter selects the winning sub-tree. This choice propagates down the tree in the form of acknowledgment and the process continues until the requesting input gets access to the shared output bus. In case of multiple event requests arriving simultaneously, a single request is selected through arbitration and the remaining requests are queued. The arbitration process selects another request from the queue when the previously selected request releases the output bus and this process continues until all the pending requests are served. Because of the queuing mechanism, events are not lost but their timing information is not preserved. A major drawback of this approach is that every request has to propagate through log_2_(*N*) stages of arbitration. As a result, throughput in this approach reduces with an increase in the number of inputs. This reduction in throughput can be significant for image sensors where multiple events often occur in close proximity. Additional details on the circuit implementation and operation can be found in Lazzaro et al. (1993) and Mahowald (1992). Improved implementation of this approach was proposed by Boahen (2004) for situations where multiple requests arrive simultaneously in close proximity. In such a case, when one input releases control of the shared bus, access is granted to the neighboring inputs without returning it to the root. This attribute of the greedy approach is illustrated by curved lines in Figure 2B. A key limitation of this approach is that both requests should arrive before access is granted by the higher-level arbiter, adding some timing requirements to the input request. A greedy tree performs very much like the original binary tree when input requests are sparse in time. Further details on the circuit implementation can be found in Boahen (2004).

Encoding the location of the selected input request is another important function of the AER encoder. The logarithmic encoder design in Mahowald (1992) and Lazzaro et al. (1993) used an arrangement where each acknowledge output drives log_2_(*N*) address lines. The design suffered from a large load on the acknowledge outputs and increased delay when scaled to a large number of input requests, affecting the overall speed and power consumption. Georgiou (Georgiou and Andreou, 2006) proposed a distributed encoding scheme to reduce the number of transistors on each acknowledge output and improve the delay on the address output. In this arrangement address bits are encoded in every stage as the token moves through the arbiter tree.

### 2.2. Ring-based arbitration

Ring-based arbitration is another approach where event-generating units, pixels, or neurons, are arranged in a ring and communicate with each other through their private servers to share the output bus. The arbitration is performed by giving one of the servers exclusive access to the shared bus. A token is used to communicate between servers and access to the shared bus is given to the server that has the token. In the case of imaging devices, pixels are arranged in a two-dimensional arrangement and each row/column of pixels is assigned a private server which then forms separate rings for row and column. The arbitration is done in two steps by selecting an individual row/column of the pixel first and then selecting a pixel within that row/column. A key benefit of this approach is improved performance when multiple events arrive in a burst.

Imam and Manohar (2011) presented an implementation, illustrated in Figure 2C, where arbitration is performed using a token ring of mutual exclusion elements and a shared counter keeps track of the token. When an input request arrives to access the shared output bus, the row server acknowledges the request if it has the token. Otherwise, it requests the token from the neighboring server and acknowledges the request after getting the token. The neighboring server passes the token if it has it or requests the token from the next neighbor. As a result, a token circulates in the ring to arbitrate and give exclusive access to the shared output bus. A major drawback of this encoding scheme is increased delay when the token has to travel a long distance for each input request, making it inefficient for sparse events. However, when a cluster of request arrives, it can scan through the section very quickly, making the design efficient. Imam and Manohar (2011) provides additional details on the circuit implementation of the token ring. An improved version based on hierarchical rings was proposed by Purohit and Manohar (2021). As shown in Figure 2D, the design consists of multiple leaf rings which are connected to each other through a higher-level token ring. The hierarchical arrangement of rings allows a single token to quickly travel from one leaf ring to another, providing fast multiplexed access to the shared output bus and reduced token travel distance for sparse events. This approach can be tailored based on the application requirements by changing the size of rings and the number of levels in the hierarchy. Further details on the design operation and circuit implementation can be found in Purohit and Manohar (2021).

Once an input request is selected through arbitration, its location is encoded and sent across the shared bus. A counter is used to track token position in ring-based arbitration. The counter value always corresponds to the current token position. When a token moves from one server to another, the counter value is incremented. As a result, the ring-based approach doesn't require a logarithmic encoder block for address encoding. In the case of hierarchical rings (Figure 2D), separate counters are used for each level of the token ring. When a token moves from one server to another, a counter that holds the token position at that level is also updated. Counter corresponding to the leaf ring provides a token position in the leaf ring and the least significant bits of the address output (Purohit and Manohar, 2021). Similarly, a higher level counter provides higher order address bits and indicates which higher level server or its associated leaf ring has the token.

## 3. Motivation

Imaging is an essential part of many scientific areas and different imaging devices are used based on their capabilities and application. Frame-based CMOS imager has been the primary choice for most applications ranging from consumer products to scientific research. Advances in CMOS fabrication technology have allowed a rapid increase in spatial and temporal resolution by increasing the number of pixels and frame rates. Unfortunately, this results in increased readout time and power consumption (Culurciello et al., 2003).

In recent years, the event-driven vision sensor has attracted a lot of attention from scientific imaging communities in academia and industry. The primary reasons for such interest are their promising properties compared to standard frame-based cameras, such as low power consumption, sub-ms latency, sparse output, and increased availability of such sensors. Despite the efficiency of data processing by the sparse output of an event-driven vision sensor, traditional computer vision algorithms cannot be readily applied because no static scene information is encoded (Brandli et al., 2014). Brandli et al. (2014) designed a vision sensor that combines an event-driven and frame-based readout circuit with a shared photodiode, allowing simultaneous output of asynchronous events and synchronous frames. A major drawback of this approach is the separate circuit for both readout modes. By having a readout circuit that can change mode and operate efficiently either as an even-driven or frame-based readout one can track fast moving objects (in event-mode) and read full images (in frame-mode) for further analysis using well-established machine vision techniques such as object recognition and classification. A similar approach aiming at combining the advantages of both readout modes is reported in Lenero-Bardallo et al. (2015). In this case, a user can toggle between Pulse Density Modulation (PDM) and Time-to-First Spike (TFS) readout mode using control bits. In PDM mode, readout corresponds to an event-based approach while TFS mode allows the user to control the number of events reported since the last Reset and generate a full image.

In the case of X-ray imaging, many spectacular advances have been made in developing new microscopy, spectroscopy, and scattering techniques but they are still limited due to the lack of capabilities in collecting simultaneous spatial information and dynamical properties at the required length and time scales (Williams et al., 2014; Tate et al., 2016; Philipp et al., 2019). One such technique is the X-ray photon correlation spectroscopy (XPCS) which is used to study the dynamics of material by analyzing temporal correlations among photons scattered by the material (Dierker et al., 1995) and requires an imaging device with good spatial and temporal resolution. An image sensor with an increased number of pixels improves spatial resolution but limits temporal resolution due to increased time for readout. An event-based readout can significantly improve readout speed, allowing the study of dynamics at a faster timescale. In contrast to XPCS, computed tomography (CT) is a technique that provides 3D structural properties of the specimen. In this technique, a 3D representation of the sample is constructed from multiple images which are recorded at different angles of rotation, thus requiring a full-frame readout capability. Similar needs for different readout capabilities in an imaging device exist in other domains as well.

Purohit and Manohar (2021) discussed a few scientific imaging applications and proposed three readout modes based on the application and sparsity of pixel data as sparse or event mode (mode-S); cluster or region-of-interest mode (mode-C); full-frame mode (mode-F). Based on the discussion in the previous section, we can see that binary tree encoding works best in mode-S by moving tokens quickly between leaf nodes. However, when multiple events occur in close proximity, the scheme performs unnecessary arbitration at each stage of the binary tree, increasing the arbitration delay. This delay gets worse with an increase in the number of events in mode-F. The greedy tree provides an improved version of the original binary tree when multiple neighboring requests arrive in a short time window by reducing unnecessary arbitration. As a result, the greedy tree performs well in mode-S and in a few cases of mode-C. The token ring-based encoding aims to improve performance in the case of mode-F and some cases of mode-C by quickly handling events sequentially. However, it suffers from increased delay in mode-S. The hierarchical token ring offer benefits of both event-based and scanning approaches. It outperforms trees in mode-F and simultaneously outperforms token rings in mode-S (Purohit and Manohar, 2021). While the encoding scheme permits automatic switching between the three modes, the overhead of maintaining information to enable this switching has a significant cost when compared to a scheme that is optimized for a single mode of operation. Thus, a new encoding scheme that can dynamically reconfigure based on readout mode would greatly improve the overall performance of imaging devices.

## 4. Field-programmable encoding

Field-programmable gate arrays or FPGAs contain an array of configurable logic blocks and re-configurable interconnect allowing them to be quickly re-programmed “in the field" to implement the desired functionality. Inspired by the programmable approach, we have designed a new field-programmable encoding scheme that can be configured to operate in different modes (greedy tree, token ring, etc.). In this section, we first present the working principle of the proposed field-programmable encoding scheme followed by the implementation details.

### 4.1. Encoding scheme

**Figure 3:**
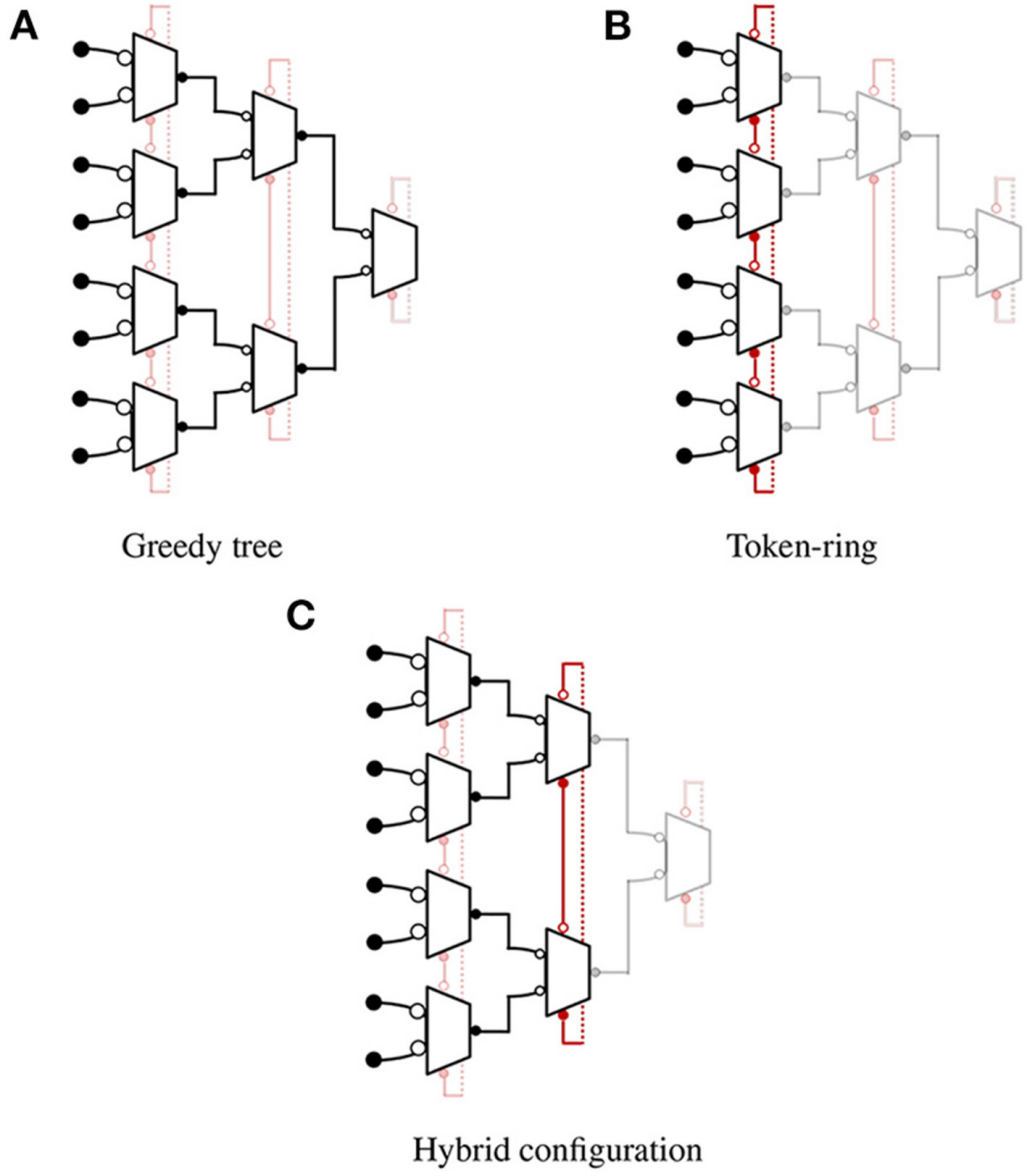
Field-Programmable AER (FP-AER) configurations. The operation of each block is set by programming configuration bit. ⊸ denotes the passive end of the channel and −• denotes the active end of the channel. **(A)** Greedy tree. **(B)** Token-ring. **(C)** Hybrid configuration.

The proposed encoding scheme, illustrated in Figure 3, is based on the greedy-tree topology from Section 2, with a modified arbiter cell. Each arbiter cell has three input channels, two output channels, and a configuration bit. The value of this configuration bit defines how access to the shared output bus is requested from the neighboring cells. As a result, the design can easily switch between event-mode (as greedy-tree) or full-frame mode (as token-ring) by programming the control bit in each stage separately. The actual implementation details are covered later. In event-mode, shown in Figure 3A, the design operates like a greedy-tree encoder where each arbiter cell requests a token from its parent cells in the higher stage when the input request arrives. Figure 3B shows how the design operates as a linear token ring in frame-mode and a single token travels through the ring to provide fast, multiplexed access to the shared output bus. The design also supports a few hybrid configurations, an example “ring of tree” configuration is shown in Figure 3C where lower stages are configured as a greedy tree and the topology ends in a token-ring at the highest level, offering configurable granularity of parallelism at lower levels for different applications. For encoding the address information, it is worth noting that the conventional address encoder and the distributed encoder by Georgiou and Andreou (2006) have roughly the same number of transistors for design containing up to 10–12 bit addresses. Since the number of pixels in an imaging device for scientific applications is typically 128/256 on each side, a conventional encoder is used to generate the address and maintain compatibility in all possible configurations.

### 4.2. Implementation

**Figure 4:**
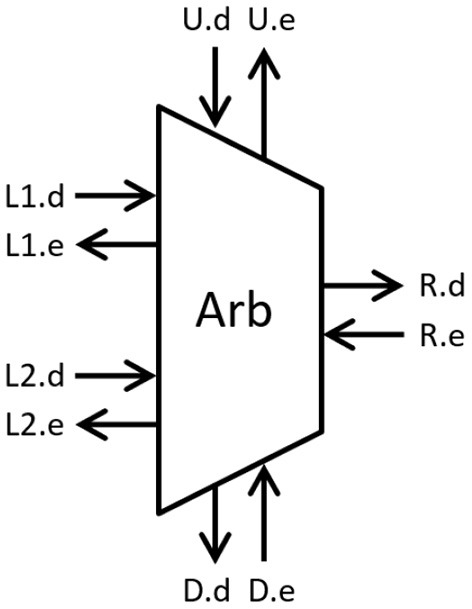
Arbitration module (request ends with “.d” and enable ends with “.e”.

The proposed design is described using Communicating Hardware Processes (CHP) and synthesized into Production Rule Set (PRS) for CMOS implementation using Martin's synthesis method (Martin, 1986) and the open-source ACT EDA flow for digital asynchronous circuits (Ataei et al., 2021). Figure 4 shows a block diagram of the arbiter with passive input communication channels *L*1 (Left1), *L*2 (Left2), and *U* (Up) and active output communication channels *R* (Right) and *D* (Down). For each communication channel, request signals are named with a “.d” suffix, and enable (inverse of acknowledge) signal end with “.e.”. The non-deterministic selection is handled using a CMOS arbiter consisting of a latch and metastability filter (Kinniment, 2008). The CHP for arbiter cell is described as:

*Arb* ≡

  * [ [ ￢*c* →

       *[ [ $\bar{L1}$ → *R*; *L*1; [$\bar{L2}$ → *L*2 | ￢$\bar{L2}$ → *skip*]; *R*

            | $\bar{L2}$ → *R*; *L*2; [$\bar{L1}$ → *L*1 | ￢$\bar{L1}$ → *skip*]; *R*

            | *U* → *skip*

         ] ]

       ⌷ *c* →

         * [ [ $\bar{L1}$ → [*b* → *skip* ⌷ ￢*b* → *D*!]; *b↑*; *L*1

               | $\bar{L2}$ → [*b* → *skip* ⌷ ￢*b* → *D*!]; *b↑*; *L*2

               | $\bar{U}$ → [*b* → *skip* ⌷ ￢*b* → *D*!]; *b↓*; *U*

            ] ]

   ] ]

In tree mode (*c* = 0), the cell performs like a greedy arbiter. When input channel *L*1 or *L*2 request access, the cell requests a token from the parent cell through channel *R* and grants access to the input *L*1 or *L*2 after receiving the token on channel *R*. When both input channels request access within a very short period, the cell arbitrates and grants access to one of the two inputs. Once the selected input returns access, the cell grants access to the second (neighboring) input without returning the token to the root. In this mode, the process ignores request on input channel *U* and do not communicate on channel *D*. Similarly, in ring mode (*c* = 1), the cell performs like a token ring and communicates with the input request channels (*L*1 and *L*2) and neighboring cells through channels *U* and *D*. When the cell receives a communication request on channel *L*1, *L*2, or *U*, it performs a four-phase handshake if it has the token (*b*). If it doesn't have the token, the cell requests a token from the cell below using a four-phase handshake on channel *D*, updates the token variable (*b*), and performs the four-phase handshake on the input channel. A dual-rail variable is used to encode the token variable.

## 5. Results and discussion

The FP-AER encoding discussed above has been implemented in a standard 65 nm bulk CMOS technology and simulated with a 1V supply at a nominal device temperature of 25 °C to verify functionality. A small capacitance was added to the output of every gate to account for parasitics and measure realistic simulation results. The SPICE simulations were performed using Synopsys HSPICE, an industry standard circuit simulation. We simulated the proposed and existing address-event encoding schemes in sparse and scan mode to evaluate their performance for event-based and frame-based imaging applications. In sparse mode, the design is configured as a greedy tree. We measured latency (request to acknowledge delay) for a randomly selected input request. Average latency from *K* such measurements, one from each input, was calculated. In the case of scan mode, the design is configured as a linear token ring. We measured the total delay for servicing all of the input requests by measuring the latency of each input from the SPICE simulation and computing the sum. To evaluate the performance of these encoding schemes, we also measured average power consumption while measuring the latency (request to acknowledge). To keep our evaluation consistent with the work done in Purohit and Manohar (2021), we designed a two-level hierarchical token ring with $\sqrt{K}$ processes in each ring. Instead of using a shared counter for ring-based designs, we used the traditional logarithmic address encoding to maintain consistency across different encoding schemes. For each encoding scheme, the latency and average power consumption are measured for 16, 64, and 256 inputs.

Based on the simulation results, listed in Table 1, we observe that tree-based topologies work best for sparse events. It handles distant input requests quickly with the help of a hierarchical tree structure. Greedy tree further improves the performance when multiple events arrive in close proximity but delay in such cases depends on the response time of the pixel and its noise characteristics (iniVation AG, 2020). In situations where input requests are sparse in time, every request has to propagate through log_2_(*N*) stages of arbitration and the greedy tree performs very much like a binary tree. In the case of full-frame mode or a situation when a burst of events arrives simultaneously, a linear token ring works best by quickly scanning through the ring. However, this approach suffers in case of a sparse event due to unnecessary token movements. The hierarchical token ring performs best in situations where the activity pattern is unknown. It handles neighboring requests by quickly scanning through leaf rings and handles sparse events by quickly moving from one leaf ring to another with the help of a hierarchical ring structure. When the activity pattern is known, FP-AER performs best by configuring it into a tree-based or ring-based topology. In sparse mode, FP-AER handles sparse events quickly by operating as a greedy tree. The difference in measured delay/power of the FP-AER compared to the greedy/binary tree in this mode is due to the extra circuit present in the design for supporting the configurability. A similar difference due to circuit complexity can be observed between binary tree and greedy tree designs. In the case of full scan mode, FP-AER quickly scans through multiple neighboring events by operating as an improved token ring. Each stage in FP-AER serves two inputs compared to one input in a token ring. As a result, FP-AER outperforms the token ring by a significant margin because a token needs to move only *K*/2 stages compared to *K* stages in the token ring.

**Table 1:** Delay and power estimation for different address-event encoding.

	**Delay**	***K* = 16**	***K* = 64**	***K* = 256**
	**(hop-count)**	**DelayPower**	**DelayPower**	**DelayPower**
**Sparse event+**				
Binary tree	2*(log_2_*K*− 1)	0.59 *ns*, 9.32 μ*W*	0.87 *ns*, 14.24 μ*W*	1.16 *ns*, 21.32 μ*W*
Greedy tree	2*(log_2_*K*− 1)	0.61 *ns*, 11.04 μ*W*	0.91 *ns*, 16.85 μ*W*	1.21 *ns*, 24.83 μ*W*
Token-ring	(*K*+ 1)/2	4.66 *ns*, 80.75 μ*W*	19.24 *ns*, 135.73 μ*W*	80.91 *ns*, 548.58 μ*W*
Hier-ring	(*H*+ *L*)/2; *K* = H*L	3.28 *ns*, 53.47 μ*W*	5.99 *ns*, 101.90 μ*W*	11.43 *ns*, 100.67 μ*W*
FP-AER	2*(log_2_*K*− 1)	0.88 *ns*, 14.88 μ*W*	1.31 *ns*, 22.67 μ*W*	1.74 *ns*, 32.57 μ*W*
**Full scan**				
Binary tree	2K*(log_2_*K*− 1)	9.43 *ns*, 0.15 *mW*	56.08 *ns*, 0.91 *mW*	297.71 *ns*, 5.45 *mW*
Greedy tree	3*K*−6	*	*	*
Token-ring	*K*	10.88 *ns*, 0.33 *mW*	45.35 *ns*, 1.43 *mW*	184.13 *ns*, 6.58 *mW*
Hier-ring	*K*+ 2*H*	17.69 *ns*, 0.55 *mW*	59.78 *ns*, 1.90 *mW*	213.37 *ns*, 7.54 *mW*
FP-AER	*K*/2	9.49 *ns*, 0.24 *mW*	39.80 *ns*, 1.06 *mW*	161.78 *ns*, 5.06 *mW*

*K*= total number of inputs, **H** and *L* are number of inputs in leaf and higher rings for Hier-ring. ^+^For ring topology, delay is measured with assumption that token always starts with initial location (at Reset) and moves to one of the Lserver.

* Delay measurement for full scan in greedy tree were not done because of the timing assumptions in event arrival and accurately measuring such delay depends on the response time of the pixel (iniVation AG, 2020), which is beyond the scope of this paper.

**Figure 5:**
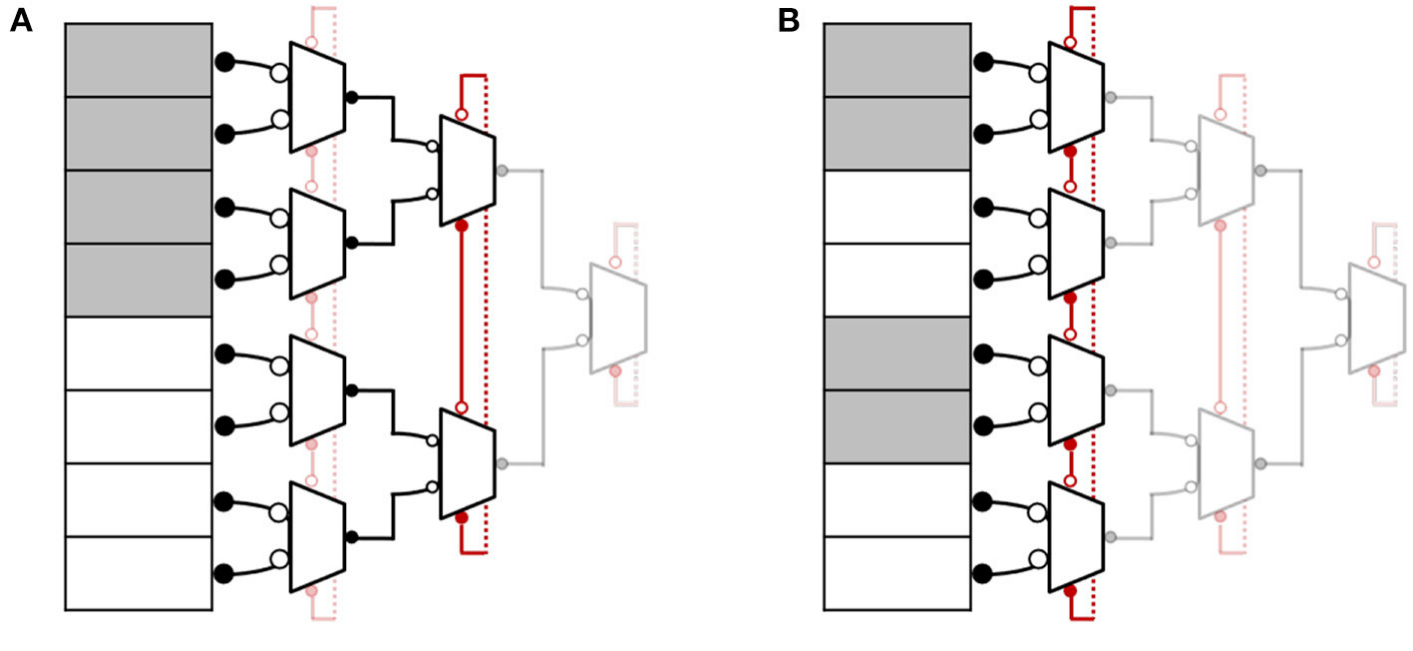
FP-AER hybrid configurations **(A,B)**.

FP-AER can also be programmed into a hybrid configuration to support different event clusters. Figure 5 illustrates some hybrid configurations to efficiently support different cluster sizes. In such configurations, the topology works as a ring of multiple small-sized trees. For example, in Figure 5A, events from the first four inputs (indicated by white boxes) are treated as a cluster and handled quickly, like a tree. After servicing this region, the token moves to the next process in the higher stage ring and serves requests from another four inputs (indicated by gray boxes). Figure 5B shows another example where a group of two inputs is treated as an event cluster and handled quickly before the token moves to the next process.

## 6. Conclusion

Frame-based readout works best in full-frame mode by quickly scanning through every pixel in the array. As the number of pixels grows, a frame-based readout suffers from high bandwidth requirements and increased power consumption. On the other hand, an event-based readout offers the best performance when a small number of pixels need to report a useful event. It suffers from increased latency and loss of timing information with an increase in the number of events. Thus, it becomes important to optimize readout design and create a mechanism where the receiver can provide feedback on data/event patterns to optimize how information is read out. We presented a new field-programmable AER encoding scheme that offers the benefits of both event-based and frame-based readout. In case of sparse events, the design operates in a tree configuration to handle events quickly. By changing the configuration bits, the design operates as a token ring to quickly scan through neighboring events. The simulation results provide a qualitative comparison of different encoding schemes and demonstrate that FP-AER performs best for all activity patterns.

## Data availability statement

The original contributions presented in the study are included in the article/supplementary material, further inquiries can be directed to the corresponding author.

## Author contributions

Both authors listed have made a substantial, direct, and intellectual contribution to the work and approved it for publication.

## Conflict of interest

The authors declare that the research was conducted in the absence of any commercial or financial relationships that could be construed as a potential conflict of interest.

## Publisher's note

All claims expressed in this article are solely those of the authors and do not necessarily represent those of their affiliated organizations, or those of the publisher, the editors and the reviewers. Any product that may be evaluated in this article, or claim that may be made by its manufacturer, is not guaranteed or endorsed by the publisher.

## Appendix

The circuit functionality is described using Communicating Hardware Processes (CHP) language and key notation of the CHP syntax are summarized below:

- Skip: No operation
- Send: *X*!*v* means send the value of *v* over channel *X*.
- Receive: *X*?*v* means receive a value on channel *X* and store it in variable *v*.
- Probe: $\bar{X}$ determines if there is a pending communication on a channel *X*
- Assignment: *a*: = *b* means assign the value of *b* to *a*.
- Sequential Composition: *S*1; *S*2 means execute statements *S*1 and *S*2 sequentially
- Parallel Composition: *S*1, *S*2 means execute statements *S*1 and *S*2 in parallel
- Deterministic Selection: [*G*1 → *S*1 [] ... [] *Gn* → *Sn*] waits until one of the guards (*G*1, *G*2...*Gn*) is *true* and then execute corresponding statement. Requires that the guards must be mutually exclusive
- Non-Deterministic Selection: [*G*1 → *S*1 | ... | *Gn* → *Sn*] is same as the Deterministic Selection except guards don't have to be mutually exclusive
- Repetition: *[*S*] infinitely repeats statement *S*.
